# Evaluating techniques for determining elasmobranch body size: a review of current methodologies

**DOI:** 10.7717/peerj.18646

**Published:** 2024-12-23

**Authors:** Ana S. Ferreira, Márcia A. Naré, Joana I. Robalo, Núria D. Baylina

**Affiliations:** 1 Oceanário de Lisboa, Lisbon, Portugal; 2 Instituto Universitário de Ciências Psicológicas, Sociais e da Vida (ISPA), Lisbon, Portugal; 3 Marine and Environmental Sciences Centre/ ARNET - Aquatic Research Network, Instituto Universitário de Ciências Psicológicas, Sociais e da Vida (ISPA), Lisbon, Portugal

**Keywords:** *Elasmobranchii*, Length/size determination, Stereo-video, Photogrammetry, Non-invasive techniques

## Abstract

There is global awareness that many species of elasmobranchs (sharks and rays) have life history characteristics that make them susceptible to overexploitation. The study of these animals is critical, as it contributes to increasing knowledge of these specimens and aids in their conservation. In particular, growth rate, age, fecundity, and size at maturity are key parameters for defining management and conservation strategies in elasmobranchs. Biometric data collection allows these parameters to be determined and considered in the evaluation of population demography. Over the last decades, several methodologies for measuring elasmobranch size have evolved, progressing from traditional capture-based methods to sophisticated, non-intrusive photographic techniques. The present review aims to understand and analyse all the existing non-invasive techniques that currently allow the collection of zoometric data in elasmobranchs and, later, to highlight the advantages and limitations of each technique, with comments on their application to fieldwork. To this end, 49 articles were selected, encompassing seven measurement techniques: photogrammetry using distance to the individual, bar photogrammetry, laser photogrammetry, stereo-DOV, stereo-BRUV, stereo-ROV, and aerial photogrammetry. Globally, the last four techniques are excellent alternatives to methods that involve animal capture or death, as they are practical, simple to use, minimally invasive, and potentially highly accurate. Each technique’s requirements related to equipment and cost, limitations, and distinctive features are presented here and summarized to guide researchers on what’s available and how to select the most appropriate for their studies.

## Introduction

The cartilaginous fishes’ subclass *Elasmobranchii* comprises sharks and rays. Compared to bony fishes, this subclass is relatively small, with 1,258 (and rising) valid known living species, and the group of rays covers more than half of this number (Ebert, Dando & Fowler, 2021; Jorgensen et al., 2022; Fricke, Eschmeyer & Van der Laan, 2024). Concerning body shape, most sharks have fusiform bodies, while all rays and some sharks have flattened bodies. The subclass *Elasmobranchii* (sharks and batoids) comprise the most common living Chondrichthyans (Ebert, Dando & Fowler, 2021) and the number of known species rises each year (Fricke, Eschmeyer & Van der Laan, 2024). Most elasmobranchs are marine and may be present at various depths. Although most species have a relatively restricted distribution, it is possible to find species from coastal areas to >2,000 m in depth across the globe and occupying a wide range of habitats (Cailliet et al., 2005; Ebert, Dando & Fowler, 2021). It is widely reported that Elasmobranchs’ life history traits (such as slow growth with late maturity, high longevity, low fertility, low productivity, and long gestation) make them extremely vulnerable to overexploitation and their ability to recover from population decreases is very limited (Dulvy et al., 2014; Dulvy et al., 2021; Ebert, Dando & Fowler, 2021). Accurate biometric data collection is therefore key to understanding the life history of each species and crucial to the application of fishing regulations and effective conservation actions to prevent their extinction (Dulvy et al., 2014; Jorgensen et al., 2022).

Biometric measurements cover a large set of features such as height, weight, fingerprints, and facial recognition (Jain & Pankanti, 2006). The shape and structure of marine organisms vary, therefore morphological measurements may vary according to different taxa (Dineshbabu et al., 2014; Kumar et al., 2017). Zoometry is the area of animal biometry that focuses on specifically collecting and analyzing the dimensions of an animal’s body. Currently, this is a widely used and growing research area with a significant demand for non-invasive techniques, which are the major focus of this study. These methods overcome the need for capture and handling, the major disadvantages of invasive techniques that are not only disruptive but potentially lethal (Bugge et al., 2011; Petso, Jamisola Jr & Mpoeleng, 2022).

In elasmobranchs, when one intends to evaluate the demography of a population to define management and conservation strategies, the most important parameters are the growth rate, age and size at maturity, fecundity, and reproductive cycle. Ideally, these data should be collected from individuals in their natural habitat; however, this is not always feasible, acknowledging that the growth patterns in *ex situ* animals differ from those collected in the natural habitat (Jañez et al., 2018; Nielsen et al., 2020; Jorgensen et al., 2022).

Capturing elasmobranchs is a complex and demanding process, which involves the use of specialized professionals and equipment, and it can be hazardous for handlers since these animals are usually wild specimens whose behavior is unpredictable and many of them are large and with powerful jaws, and poisonous spines. Given these factors, various methodologies for measuring elasmobranchs have been developed over time to reduce the risks to both handlers and animals (Klimley & Brown, 1983; Dunbrack, 2006; Delacy et al., 2017; Raoult et al., 2019).

The present review is an innovative study that aims to assess the non-invasive techniques available for measuring elasmobranchs, providing guidance on the most advantageous and reliable ones to use, according to the research objectives. The main goal is to describe each method, highlighting its advantages, requirements, limitations, and applications.

## Survey Methodology

This review was based on a literature search using Google Scholar and ISI Web of Knowledge databases. The following expressions/keywords, were searched for: “simple field method length measurement”, “size measurement of elasmobranch”, “measuring elasmobranch”, “size measurement of sharks”, “size estimates in elasmobranch”, “measuring skates body size”, “length measurement skates”, “remote measurement elasmobranch”, “stereo systems to measure elasmobranch length”, “stereo systems to remote measurement of sharks”, “stereo-video to assess body size in elasmobranch”, “stereo systems to remote measurement elasmobranch”, “baited remote systems in sharks”, “BRUV length Batoidea”, “BRUV for measuring sharks”, “ROV for measuring elasmobranchs”; “using diver operated stereo-video system to measure”, “using diver operated stereo-video system to measure sharks”, “stereo-DOV for remote measurement of elasmobranch”, “aerial photogrammetry for measuring elasmobranchs“, “the use of drones for elasmobranch measurements“, “remote techniques for measuring sharks and rays “, “parallel lasers remote measurements in elasmobranch”, “determination of size “laser photogrammetry””, “using lasers to measure elasmobranch”, “laser photogrammetry in elasmobranch”, “laser photogrammetry in sharks”, “measurement with parallel lasers in sharks”, “measuring sharks using bar photography”, “measuring sharks body size with bar”. Additionally, an analysis of the bibliography of the assembled articles was undertaken to increase the number of relevant articles to include in this revision.

Bibliographic research was restricted to peer-reviewed articles that referred to body measurement methods and techniques specifically applicable to *Elasmobranchii*. Papers on other species, measurement techniques by “eye”, capture and/ or death of individuals, works focused on population counts, species abundance, behavioral studies, and articles that rely exclusively on the photo-identification of individuals were excluded. Microsoft Office Excel Software was used to organize the data during the bibliographic collection. The seven photogrammetry techniques analyzed are presented and briefly described, and for each one, a summary of its requirements and distinctive features (positive and negative) are presented in Table 1. The selected articles’ information was organized in a table where the technique, title, authors, publication date, species under study, reported error when present, keywords, and link to the article were included. This information was subsequently compiled to present the technique used in each study, the authors and publication date, and the main outcomes, mentioned by its authors (Table 2).

**Table 1:** Name and a brief description of the non-invasive photogrammetry techniques analyzed for measuring elasmobranchs. The equipment requirements and distinctive features (positive and negative) for each technique are presented.

**Technique**	**Brief description**	**Equipment requirements**	**Distinctive positive features**	**Distinctive negative features**
Photogrammetry using distance to the individual	It involves estimating the size and shape of elasmobranchs by measuring the distance from the camera to the individual. It requires precise distance measurement tools to ensure accurate scaling of the images. Distances to objects on the sea surface can be calculated from photographic images by measuring the angle of dip from the horizon to the object from images taken from a known height with a calibrated lens.	Minimal equipment (camera, reference object). Low cost.	Very simple technique. Minimal requirements. Ideal for species near the surface.	Limited to surface-near individuals. Partial visibility restricts measurements. Body bending affects accuracy.
Bar photogrammetry	This technique uses a calibrated bar of known length placed in the camera’s field of view, parallel to the animal, serving as a reference scale for measuring the size of the elasmobranchs captured in the images.	Few equipment needed (camera, reference bar, mounting system). Reduced cost.	Simple technique. Few requirements. Ideal for species that approach and stay parallel to the bar.	Requires individuals to be parallel and close to the bar. Increased error due to passage through various media.
Laser photogrammetry	Utilizes parallel and calibrated laser pointers projected onto the elasmobranchs, forming a scale of known distance on the animal’s body. The spacing between the laser points provides a reference for accurate size measurements. Images should include the whole animal perpendicular to the camera.	Requires laser pointers or rangefinders to project laser lines. Lasers must be parallel and calibrated. Software needed for image processing and measurements.	Low associated cost. Low impact on individuals. Various free measurement software available. Low error percentage if precautions are followed.	Requires parallel lasers and immobile equipment to minimize error. Strict image collection requirements (perpendicular, 2D). Susceptible to environmental conditions affecting laser visibility.
Stereo-DOV	Involves divers using calibrated stereo camera systems to record video of elasmobranchs. The two cameras capture images from slightly different angles. While the measurements are linear, this setup enables three-dimensional estimates of the animals.	Specific hardware and software needed; considerable cost. Low-cost options available. Diver required.	Selective sampling due to diver maneuverability. Allows targeting specific species’ populations.	Depth limitation due to diver’s capabilities.
Stereo-BRUV	This method employs stereo camera systems mounted on usually baited frames and deployed. The bait attracts elasmobranchs into the field of view, enabling the capture of stereoscopic images for size and behaviour analysis. The system is similar to the stereo-DOV but can operate at greater depths and for longer time durations than those possible when using divers.	Specific hardware and software; considerable cost. Low-cost options available. Typically requires BRUV deployment and collection by boat.	Remote sampling capability. Enables sampling in deep locations without divers.	Bait presence can be disruptive. May bias behavior and abundance results.
Stereo-ROV	Uses stereo cameras mounted on remotely operated vehicles to capture three-dimensional images of elasmobranchs in their natural habitat, providing accurate measurements and detailed observations without diver intervention. It can operate far deeper than the stereo-DOV and it is highly mobile unlike the stereo-BRUV.	Specific hardware and software; considerable cost. Reduced-cost options with significant limitations. Requires higher maneuvering skills. Equipment maintenance costs.	Versatile for multiple studies (size structure, behavior, 3D modeling). Operates at great depths. Advanced industry ROVs useful for combined scientific campaigns. Opportunistic images can hold significant scientific value.	Requires specific deployment and operation protocols. Sampling limitations due to protocols. Sound and lights may deter fish.
Aerial photogrammetry (UAV)	Involves unmanned aerial vehicles (drones) equipped with cameras to capture images of surface-swimming elasmobranchs. Ground sampling distance could be used as a scale. It surveys extremely large areas (in the order of Km) from above.	Specific hardware and software; considerable cost. Low-cost options available (short battery life). Requires known length object as scale or advanced altitude sensors.	Minimal impact on measured animals. Suitable for simple, accurate, and affordable measurements. Uses small, commercially available drones.	Allows measurements only near the surface. Requires calm water conditions. Needs good surface visibility.

**Table 2:** Summary of the 49 articles analyzed. Publication details: authors, year of publication, applied technique, species studied and main outcomes of each study.

**Publication**	**Species studied**	**Main outcomes**
Lacey et al. (2010) ^1^	*Cetorhinus maximus*	Large sample measured and systematic bias related to body flexing less than 10%.
Govender, Kistnasamy & Van der Elst (1991) ^2^	*Carcharias taurus*	Robust technique for size estimation.
Araujo et al. (2014) ^3^	*Rhincodon typus*	Effective and efficient technique to measure large sharks.
Bansemer & Bennett (2009) ^3^	*Carcharias taurus*	Measuring females allowed detailed monitoring of females’ reproductive cycle.
Barker & Williamson (2010) ^3^	*Carcharias taurus*	Measured individuals added individuals to the data base. Lasers proved to be accurate.
Deakos (2010) ^3^	*Mobula alfredi*	Effective for *M. alfredi* measurement, with the conversion of disc length to disc width by applying a ratio function.
Deakos (2012) ^3^	*Mobula alfredi*	Allowed the measurement of fully stretched individuals, and evaluated multiple factors of *M. alfredi’s* reproductive ecology.
Devine, Wheeland & Fisher (2018) ^3^	*Somniosus microcephalus*	The males and females observed and measured may all be sexually immature in the sampled areas.
Guttridge et al. (2017) ^3^	*Sphyrna mokarran*	Measurements were used to assess the life stage and maturity of sharks, providing further insights into the observed philopatric behaviour of *S. mokarran.*
Hearn et al. (2016) ^3^	*Rhincodon typus*	Tagged *R. typus* females exhibited higher length estimates than those reported in previous studies
Jeffreys et al. (2013) ^3^	*Rhincodon typus*	The device demonstrated ease of calibration, robustness, and reliability, with reduced costs, and safely provided accurate measurements in water.
Leurs et al. (2015) ^3^	*Carcharodon carcharias*	This technique effectively evaluated species size and growth, showing superiority over visual estimates, and enhancing photo-identification using dorsal fins.
O’Connell & Leurs (2016) ^3^	*Sphyrna mokarran*	Laser photogrammetry was effective for *S. mokarran* measurements, aiding future studies on growth rates and site fidelity.
Perry et al. (2018) ^3^	*Rhincodon typus*	Laser and tape measure techniques had lower errors and were more accurate than visual estimates.
Rezzolla, Boldrocchi & Storai (2014) ^3^	*Sphyrna lewini*, *Carcharhinus amblyrhynchos*	Low-cost, continuous, non-invasive equipment effectively monitored species’ ecology, measuring seven specimens and producing substantial data in challenging environments over a brief study period.
Rogers, Cambiè & Kaiser (2017) ^3^	*Scyliorhinus canicula*	This study was the first to use allometry changes to detect shark maturity, with laser measurements proving accurate.
Rohner et al. (2011) ^3^	*Rhincodon typus*	Laser photogrammetry was economical and accurate for measuring *R. typus* in natural habitats, surpassing visual methods.
Rohner et al. (2015) ^3^	*Rhincodon typus*	The technique enhanced size estimation accuracy for whale sharks compared to visual methods.
Rowat et al. (2011) ^3^	*Rhincodon typus*	Laser photogrammetry provided more accurate length assessments than visual or tape measurements by divers.
Delacy et al. (2017) ^4^	*Carcharhinus longimanus*	Simple equipment and software yielded accurate measurements, with 2D calibration proving as effective as 3D calibration.
Goetze et al. (2018) ^4^	*Aetobatus ocellatus*, *Carcharhinus amblyrhynchos*, *C. melanopterus*, *Negaprion acutidens*, *Taeniura lessoni*, *Triaenodon obesus* and *Urogymnus granulatus*	This study measured over 100 sharks and quantified shark and ray abundance, biomass, and diversity in the Solomon Islands.
Klimley (1987) ^4^	*Sphyrna lewini*	*S. lewini* females were found to mature at a larger size and grow more rapidly than males.
Klimley & Brown (1983) ^4^	*Sphyrna lewini*	The portable measurement method with two cameras was effective for *S. lewini* size and position determination.
May et al. (2019) ^4^	*Carcharodon carcharias*	Visual estimates by experienced observers were validated for *C. carcharias* studies although stereo photographs for measurements were minimally addressed.
Meekan et al. (2020) ^4^	*Rhincodon typus*	Body length measurements were used to estimate growth rates and different asymptotic total lengths between males and females.
Salinas-de León et al. (2016) ^4^	Miscellaneous (not all species observed and measured were specified, nor whether they were analyzed by the DOV or by visual census).	The first quantitative fish survey using stereo-video improved length estimate accuracy and highlighted the ecological value of Darwin and Wolf Islands.
Sequeira et al. (2016) ^4^	*Rhincodon typus*	The stereo system accurately estimated *R. typus* length.
Acuña Marrero et al. (2017) ^5^	*Galeocerdo cuvier*	Large sharks (>200 cm TL) frequently visited green sea turtle nesting beaches at night, while smaller sharks rarely did and were active elsewhere during the day.
Asher, Williams & Harvey (2017) ^5^	* *Carcharhinus galapagensis* and *Triaenodon obesus*	BRUV offered benefits over diver surveys for measuring sharks and assessing predator populations at greater depths. The studied areas were key for both juveniles and adults.
Asher, Williams & Harvey (2019) ^5^	* *Carcharhinus galapagensis* and *Triaenodon obesus*	There were several inconsistencies between predator length distributions generated from different methods. BRUVS might provide a less biased predator length-frequencies, given they can sample a greater depth range without the confounding size-estimation discrepancies noted with divers
Dunbrack (2006) ^5^	*Hexanchus griseus*	The technique was relatively insensitive to camera alignment changes, providing accurate length measurements for demersal species.
Dunbrack & Zielinski (2005) ^5^	*Hexanchus griseus*	The minimally invasive, remote technique allowed length measurements of 35 free-swimming *H. griseus.*
Goetze & Fullwood (2013) ^5^	*Carcharhinus albimarginatus*, *C. amblyrhynchos*, *C. melanopterus*, *Stegostoma fasciatum* and *Triaenodon obesus*	The study demonstrated the positive impact of a Marine Reserve on reef shark biomass, showing BRUVs’ suitability for size estimation sampling.
Harasti et al. (2016) ^5^	*Carcharodon carcharias*	BRUVs effectively allowed monitoring *C. carcharias’* juvenile size and abundance over 24 months.
Harasti et al. (2019) ^5^	*Carcharodon carcharias*	BRUVs demonstrated success in monitoring *C. carcharias* juveniles’ length, showcasing practicality for long-term assessments.
Letessier et al. (2013) ^5^	Not all measured species are specified.	The system offered a low-cost, efficient tool for fast, non-extractive size estimation and monitoring of pelagic fish and shark populations.
Lewis et al. (2023) ^5^	*Notorynchus cepedianus*	The system had a small error and produced accurate, repeatable measurements.
Pimentel et al. (2019) ^5^	*Carcharhinus falciformis, C. galapagensis*, *C. perezi*, *Galeocerdo Cuvier*, *Ginglymostoma cirratum* and *Sphyrna lewini*	BRUV was a useful complement to other non-invasive methods to measure sharks.
Pinte et al. (2020) ^5^	*Centrophorus harrissoni*, *C. squamosus*, *Centroscymnus owstonii*, *Dalatias licha*, *Deania calcea*, *Etmopterus granulosus*, *E. molleri* and *Proscymnodon plukenti*	First *in-situ* study of deep-sea species’ swimming speeds. 253 speed and 135 size measurements from 28 species were collected.
Ryan et al. (2015) ^5^	*Carcharhinus albimarginatus*, *C. amblyrhynchos*, *C. brevipinna*, *C. plumbeus*, *C. obscurus*, *Furgaleus macki*, *Galeocerdo cuvier*, *Hemitriakis falcata*, *Heterodontus portusjacksoni*, *Mustelus antarcticus*, *Nebrius ferrugineus*, *Negaprion acutidens*, *Parascyllium variolatum*, *Sphyrna lewini* and *Triaendon obesus*	The study amassed a large dataset on species’ cruising speeds and sizes, proving BRUV useful for body size and speed measurements.
Santana-Garcon et al. (2014a) ^5^	* *Carcharhinus limbatus*, *C. plumbeus*, *Carcharhinus spp.*, *Galeocerdo cuvier*, *Rhizoprionodon acutus*, *Sphyrna lewini* and *S. mokarran*	Pelagic BRUVs provided a non-lethal alternative for shark sampling, with size estimates comparable to longline methods.
Santana-Garcon et al. (2014b) ^5^	* *Carcharhinus limbatus*, *C. plumbeus*, *Carcharhinus spp., and Galeocerdo cuvier*	The robust, non-destructive method allowed standardized studies of fish assemblages, providing permanent species records, measurements, and behavioral observations.
Tickler et al. (2017) ^5^	*Carcharhinus albimarginatus*, *C. amblyrhynchos*, *C. melanopterus*, *Galeocerdo cuvier*, *Nebrius ferrugineus*, *Sphyrna lewini*, *S. mokarran* and *Triaenodon obesus*	Grey shark lengths differed significantly between locations and shark abundance is related to planktivore biomass.
Yon et al. (2020) ^5^	* *Carcharhinus melanopterus*, *Chiloscyllium punctatum*, *Hemitriakis falcata*, *Nebrius ferrugineus,Negaprion acutidens* and *Triaenodon obesus*	It was possible to measure 68% ( *n* = 58) of the observed sharks. The combined lengths of all shark species did not differ among sampled sites.
Vögler et al. (2022) ^6^	*Hexanchus griseus*	ROVs with HD cameras and laser pointers were used to determine shark length at 320m depth.
McLean et al. (2019) ^6^	*Rhincodon typus, Mobula birostris*, *Taeniurops meyeni*, *Carcharhinus amblyrhynchos* and *Triaenodon obesus*	This technique allowed observation and measurement of threatened elasmobranch species while performing industry-specific tasks*. R. typus* was recorded on 5 out of 18 days, and 2 males and 2 females were identified and measured.
Oleksyn et al. (2020) ^7^	*Bathytoshia brevicaudata*	UAVs offered a low-cost, non-invasive method to collect high-resolution marine animal size estimates, with long tracking capabilities across tidal stages.
Setyawan et al. (2022) ^7^	*Mobula alfredi*	The study demonstrated an accurate, affordable method to measure surface-feeding *M. alfredi*, with minimal animal impact and strong linear correlation in dimensions.
Whitehead et al. (2022) ^7^	*Rhincodon typus*	The first UAV study measuring whale sharks from aerial imagery, providing a viable method for obtaining biological information and tracking live individuals over time.

**Notes.**

1 Photogrammetry using distance to the individual.

2 Bar photogrammetry.

3 Laser photogrammetry.

4 Stereo-DOV.

5 Stereo-BRUV.

6 Stereo-ROV.

7 Aerial photogrammetry.

* Several species were analysed in this study; only the ones cited were measured.

## Results and Conclusions

This review is the first to identify the most common non-invasive techniques used to measure elasmobranchs to date. The 49 articles selected for further analysis allowed the identification of seven techniques reported to measure elasmobranchs: photogrammetry using distance to the individual; bar photogrammetry; laser photogrammetry; diver operated stereo-video system (stereo-DOV), stereo baited remote underwater video system (stereo-BRUV), stereo-video remoted operated vehicle (stereo-ROV) and aerial photogrammetry using unmanned aerial vehicles (UAVs, commonly mentioned as drones). Still, it was possible to verify that from these, only five techniques are currently being used. Laser photogrammetry, stereo-DOV, stereo-BRUV, stereo-ROV, and aerial photogrammetry are more accurate, allowing for larger and broader sampling but also requiring a considerable cost in terms of equipment and time to process the images.

The studies integrated in the present review were published between 1983 and 2023 and involved 42 shark and seven ray species. This finding corroborates previous studies indicating that elasmobranch species’ assessments are heterogeneously distributed. Sharks, particularly Lamniformes and Carcharhiniformes, are much more extensively assessed compared to batoids and less charismatic species (Dulvy et al., 2014; Flowers, Heithaus & Papastamatiou, 2020; Jorgensen et al., 2022). The complete list of papers analyzed can be found in the references section. Table 1 presents a brief description of each technique, the main equipment requirements, and distinctive features (positive and negative). Table 2 summarizes the 49 analyzed articles. Inspection of Table 2 revealed that *Rhincodon typus* (Smith, 1828) was the most frequently studied species (12 studies). For the rays, *Mobula alfredi* (Krefft, 1868) was the most cited, in three articles. Of all the studies considered, only two were carried out under controlled conditions, Govender, Kistnasamy & Van der Elst (1991) studied *Carcharias taurus* (Rafinesque, 1810) and Delacy et al. (2017) investigated *Carcharhinus longimanus* (Poey, 1861). Regarding the techniques used, one article published in 2010 (Lacey et al.) referred to photogrammetry using the distance to the individual, one used bar photogrammetry (Govender, Kistnasamy & Van der Elst, 1991), 17 applied laser photogrammetry, eight the stereo-DOV, 17 the stereo-BRUV, two the stereo-ROV and three used aerial photogrammetry.

### Photogrammetry using distance to the individual and bar photogrammetry

Photogrammetry using distance to the individual involves estimating the size and shape of elasmobranchs by measuring the camera-to-subject distance. It requires precise distance measurement tools to ensure accurate scaling of the images. Distances to objects on the sea surface can be calculated from photographic images by measuring the angle of dip from the horizon to the object in images taken from a known height with a calibrated lens. This method is subject to common sources of error including lens distortion, angle and distance inaccuracies, and environmental influences (Gordon, 2001; Lacey et al., 2010). In the study by Lacey et al. (2010), several key sources of error were identified. The swell-induced vertical displacement, the vertical movement of both the vessel and shark due to swell, was a significant factor. A simulation study indicated that a 0.5 m swell produced a standard deviation (SD) of error at 0.068 relative to the distance, and for more typical conditions with a 0.25 m swell, the SD was calculated to be 0.034. The shark body flexing, especially during swimming, introduced inaccuracies in length measurements. This flexing was modeled as a sine wave (based on dogfish swimming) with a flex amplitude resulting in a mean length underestimation of approximately 5%. Errors from lens calibration inconsistencies and variations in the shark’s body angle relative to the camera added further variance to the measurements. Variation in measurements within image clusters, assumed to reflect measurement error from the images themselves, yielded a mean SD of 0.09 m. Combined with the model of body flexing and swell effects, the overall error was approximated at 10% of the shark’s total length.

Bar photogrammetry uses a calibrated bar of known length placed in the camera’s field of view, parallel to the animal, serving as a reference scale for measuring the size of the elasmobranchs captured in the images. Errors associated with this method are introduced by refraction and photographic alignment. In a study by Govender, Kistnasamy & Van der Elst (1991), observations revealed that light refraction through water, air, and tank glass led to a consistent overestimation of shark length by approximately 5%. To correct for this, a compensation factor was applied to measurements. In the mentioned study, measurements were taken as sharks swam closest to a reference window, with the alignment minimized for distortion. By capturing images from this closest point (50 cm from the window), distortions due to angle or depth changes were reduced.

Both mentioned techniques require little equipment and have very low cost, and they rely heavily on stable calibration and controlled imaging angles to minimize distortions and measurement biases. They additionally require the animal to be at the surface (in the first method), and parallel and close to the bar (in the latter), which significantly limits its application. Although being the first steps towards developing photogrammetry methodologies with low-cost applications, these two techniques are obsolete and less efficient compared to modern digital and automated methods and, therefore, are no longer in use.

### Laser photogrammetry

Laser photogrammetry utilizes parallel and calibrated laser pointers projected onto the elasmobranch, forming a scale of known distance on the animal’s body. The spacing between the laser points provides a reference for accurate size measurements. Images should include the whole animal perpendicular to the camera (Rohner et al., 2011; Araujo et al., 2014).

Laser photogrammetry for measuring sharks and rays can be subject to several notable errors. One primary error source is image distortion due to water refraction, particularly if there is any misalignment between the lasers and the camera setup. This refraction often causes measurement overestimates, though correction factors can be applied to mitigate these distortions. The technique’s accuracy is also affected by the animal’s orientation and movement relative to the camera, as even minor deviations from perpendicular can introduce errors of <3% (*e.g.*, Barker & Williamson, 2010; Leurs et al., 2015) and up to 5% (*e.g.*, Bansemer & Bennett, 2009; Rogers, Cambiè & Kaiser, 2017). Consistent parallel alignment of lasers is crucial; misalignment increases error in size estimates, especially at varied distances (Jeffreys et al., 2013; Leurs et al., 2015). Field calibration, such as using fixed measurement grids or objects, can reduce these errors and improve measurement reliability for in-water applications like monitoring shark and ray morphometry and growth rates (Perry et al., 2018).

The limitations of laser photogrammetry, such as the requirement for individuals to be perpendicular to the device, at the same level as the observer, and without body flexion, make sampling complex and time-consuming to obtain high-quality images for measurement. Overcoming these challenges necessitates investing in expensive equipment to ensure the lasers’ distance, parallel alignment, and advanced image processing software. This investment reduces the primary advantage of this technique, which is its low cost. New technologies provide a three-dimensional and more realistic approach thus, laser photogrammetry will probably be replaced with more advanced methods (Pulido Mantas et al., 2023). No article using this technique was found published in the last five years, corroborating this assertion.

### Stereo-DOV

Based on the articles and techniques described, and considering their advantages and disadvantages, it is evident that stereo-DOVs have a vast field of application when the sampling depth is within a diver’s reach and the diver’s presence has minimal impact on the target species. The first use of Stereo-DOV was reported in 1983 by Klimley & Brown (1983), and this technique is currently widely applied. It comprises two synchronized cameras mounted at a fixed distance and angle apart, capturing stereoscopic footage that enables precise measurements of length and size without physically handling the animals (Harvey et al., 2010). By allowing researchers to calculate distance and depth within the video frame, stereo-DOVs can effectively gather morphometric data, monitor species behavior, and assess elasmobranch population structure (Langlois et al., 2010). Stereo-DOV systems, while powerful for underwater measurements, face several limitations and sources of error. One common issue is related to camera alignment and calibration stability, which can shift due to environmental conditions like temperature and pressure changes, affecting measurement accuracy (Harvey et al., 2003). Movement and orientation of the target organism also contribute to error, as accurate length measurements require the fish to be within a specific alignment range relative to the camera. Additionally, parallax issues arise if cameras are not perfectly synchronized, particularly with fast-swimming species, leading to slight inaccuracies (Harvey & Shortis, 1996). Furthermore, variations in water clarity and lighting can degrade image quality, making it harder to achieve precise measurements in natural, variable underwater conditions. Studies using stereo-DOV systems for measuring elasmobranchs report variable error rates, often influenced by environmental conditions and species behavior. For instance, error percentages for fish length measurements can be low, with some studies reporting mean errors around 1–3% when fish are positioned within an optimal range of 3–5 m from the camera and orientation is carefully managed. In such conditions, errors typically stay below 5% for most species (Harvey et al., 2003; Delacy et al., 2017). Accuracy can decrease with increased distance from the camera and fish movement, reaching up to 10% error under suboptimal conditions, such as low visibility or rapid swimming, which affects stereo alignment and measurement precision (Langlois et al., 2010). The presence of divers restricts stereo-DOV systems to depths accessible with SCUBA equipment, typically up to 30–40 m, depending on location and environmental conditions. This constraint limits their use in surveying deeper habitats and elasmobranch populations that occur beyond safe diver-operable depths (Langlois et al., 2010).

### Stereo-BRUV

This method employs stereo camera systems, typically mounted on baited frames, which are deployed either downward or horizontally to lure elasmobranchs into the cameras’ field of view. By capturing stereoscopic images, this setup facilitates accurate size measurements and behavioral observations of these species. Unlike stereo-DOV systems, it can be utilized at greater depths and for prolonged durations, as it operates independently of divers. However, Stereo-BRUV may yield biased results, particularly concerning abundance when analyzed alongside body measurements, due to the use of bait and potential disturbances from additional lighting. Sample limitations mirror those outlined in the previous section on stereo-DOV, with several studies indicating challenges arising from individuals not being fully visible in the cameras (Ryan et al., 2015; Pinte et al., 2020) or, in more severe cases, the cameras becoming partially obstructed after deployment (Goetze & Fullwood, 2013; Yon et al., 2020).

### Stereo-ROV

Stereo-ROV refers to a stereo imaging system mounted on an ROV that captures high-resolution, three-dimensional images of elasmobranchs in their natural habitat. This technology enables researchers to accurately measure the size and assess the behavior of these species *in situ*. By utilizing dual cameras positioned at specific angles, stereo-ROVs provide precise stereoscopic measurements, allowing for enhanced data collection in environments that may be challenging or unsafe for divers to access (McLean et al., 2019).

A similar situation to stereo-BRUV applies to stereo-ROV, where accurate measurements require a higher investment in equipment and training. These robust devices are mostly designed to sample at greater depths and according to the literature, usually for additional survey goals than biometrics, like visual assessments of fish assemblages (Sward, Monk & Barrett, 2019) or industry inspections and opportunistic sightings (Todd et al., 2020). The extremely high costs of ROVs are unlikely to lead to their adoption over stereo BRUVs and DOVs, which are considerably more affordable. This makes the latter the preferred choice for student projects and other initiatives with limited budgets that do not require extensive deep sampling (Schramm et al., 2020).

### Aerial photogrammetry

Aerial surveys using drones sample extremely large areas, require considerable altitude, water clarity, and clear skies, and analyze animals close to the surface. This recent technique is being used to assess species that best fit these requirements (mostly marine mammals) but has mainly been applied to studies of abundance, distribution, behavior, and shark-human interactions (Oleksyn et al., 2020; Setyawan et al., 2022). In 2021, Butcher et al. (2021) published a review focusing on how drones are currently being used for shark monitoring, estimating total lengths, and filling knowledge gaps around fundamental shark behaviors or movements, social interactions, and predation across multiple species and scenarios. This technique has also been used with fair results to estimate elasmobranchs’ length (*e.g.*, Whitehead et al., 2022 for *R. typus* estimates) using a known length object as a reference (one-meter-long pole) and previously testing the methodology measuring a known size object (paddleboard) at different altitudes, reporting <2% errors. However, estimates of UAV altitude and aquatic animal depth, if uncorrected, will lead to inaccurate measurements despite high-resolution images or video frame grabs (Rex et al., 2024). Aerial photogrammetry requires specialized, costly equipment to achieve precise length measurements of elasmobranchs, making it a resource-intensive approach for accurate data collection.

Although not 100% accurate, the methodologies listed represent excellent alternatives to traditional methods, involving the capture or killing of individuals (*e.g.*, Merly et al., 2019). It is noteworthy that these approaches can be used for various purposes such as studies of population’s abundance or structure (Barley, Meekan & Meeuwig, 2017; Tuya et al., 2021), photo-identification (Bansemer & Bennett, 2009; O’Connell & Leurs, 2016; Moreno, Solleliet-Ferreira & Riera, 2021), growth rates (*e.g.*, Perry et al., 2018), reproductive ecology (*e.g.*, Deakos, 2012), and morphometric traits (Carrier, Heithaus & Simpfendorfer, 2018; Rastoin-Laplane et al., 2020). They have been applied to other marine species, *e.g.*, blue whales (Durban et al, 2016), pilot whales (Wong & Auger-Méthé, 2018), , bottlenose dolphins (Aswegen et al., 2019) and various teleost fish (Harvey et al., 2003; Schramm et al., 2020).

The initial costs associated with video techniques are often considered high compared with more traditional biometric data collection methods (especially if the cost of animal capture in more traditional methods is excluded) and may probably be the cause of their scarce application in some research fields. However, several authors mentioned that the costs may be offset by a reduction in field time, staff needed, observer bias, and the provision of a permanent visual record that allows archived images to be revisited and compared against time series data for the detection of spatial and temporal variability of a vast range of species (*e.g.*, Bennett et al., 2016; Langlois et al., 2020). Moreover, reducing cameras’ costs due to advances in technology and monitoring procedures made the use of video techniques more affordable to researchers in recent decades (Bennett et al., 2016; Delacy et al., 2017; Pulido Mantas et al., 2023). The stereo-DOV technique in particular has potential for use in managed environments such as aquariums, where many of the issues described become less relevant: good visibility of the individuals, desensitization of animals to their environment and the diver’s presence, reducing flight risk, and the availability of technical and financial resources, although results extrapolation is limited (Ferreira et al., 2024). Non-invasive techniques for length estimates are extremely important for elasmobranch studies as explored in the present review. They represent accurate, accessible, and versatile methodologies that contribute effectively to elasmobranchs’ management and conservation.

## Additional Information and Declarations

### Data Availability

The following information was supplied regarding data availability: This is a literature review.
